# Infection and Genomic Properties of Single- and Double-Stranded DNA *Cellulophaga* Phages

**DOI:** 10.3390/v17030365

**Published:** 2025-03-03

**Authors:** Cristina Howard-Varona, Natalie E. Solonenko, Marie Burris, Marion Urvoy, Courtney M. Sanderson, Bejamin Bolduc, Matthew B. Sullivan

**Affiliations:** 1 Department of Microbiology, The Ohio State University, 484 W 12th Ave, Columbus, OH 43210, USA; howard-varona.2@osu.edu (C.H.-V.); solonenko.2@osu.edu (N.E.S.); burris.183@osu.edu (M.B.); urvoy.1@osu.edu (M.U.); sanderson.107@buckeyemail.osu.edu (C.M.S.); bolduc.10@osu.edu (B.B.); 2 Center of Microbiome Science, Ohio State University, Columbus, OH 43210, USA; 3 Department of Civil, Environmental and Geodetic Engineering, The Ohio State University, 2070 Neil Ave, Columbus, OH 43210, USA; 4 Center for RNA Biology, The Ohio State University, 484 W 12th Ave, Columbus, OH 43210, USA

**Keywords:** virus, bacteriophage, flavophage, ssDNA phage, dsDNA phage, bacteria, *Cellulophaga*, marine, genome, infections

## Abstract

Bacterial viruses (phages) are abundant and ecologically impactful, but laboratory-based experimental model systems vastly under-represent known phage diversity, particularly for ssDNA phages. Here, we characterize the genomes and infection properties of two unrelated marine flavophages—ssDNA generalist phage phi18:4 (6.5 Kbp) and dsDNA specialist phage phi18:1 (39.2 Kbp)—when infecting the same *Cellulophaga baltica* strain #18 (Cba18), of the class *Flavobacteriia*. Phage phi18:4 belongs to a new family of ssDNA phages, has an internal lipid membrane, and its genome encodes primarily structural proteins, as well as a DNA replication protein common to ssDNA phages and a unique lysis protein. Phage phi18:1 is a siphovirus that encodes several virulence genes, despite not having a known temperate lifestyle, a CAZy enzyme likely for regulatory purposes, and four DNA methyltransferases dispersed throughout the genome that suggest both host modulation and phage DNA protection against host restriction. Physiologically, ssDNA phage phi18:4 has a shorter latent period and smaller burst size than dsDNA phage phi18:1, and both phages efficiently infect this host. These results help augment the diversity of characterized environmental phage–host model systems by studying infections of genomically diverse phages (ssDNA vs. dsDNA) on the same host.

## 1. Introduction

Viruses are the most abundant entities on Earth [1,2], including in the oceans [3] where most are thought to be bacteriophages (i.e., phages) infecting bacteria [2,3,4] that outnumber ~10-fold their bacterial hosts [5,6,7,8,9]. At any given time, marine viruses infect 20–40% of surface microbes daily, with 10^23^ infections occurring each second [7,10,11]. These infections have myriad impacts on cellular functions [12,13,14,15,16,17,18,19,20,21], nutrients [2,22], microbial evolution and mortality [4,11,13], and cross-kingdom interactions [20,23]. Additionally, machine learning and statistical modeling of global ocean plankton inventories suggests that the abundance of viruses, more so than that of prokaryotes or eukaryotes, best predicts global ocean carbon flux from surface to deep waters [24,25,26,27]. Experimentally, numerous studies now reveal that virus-infected cells (i.e., virocells) completely reprogram host biomolecules (e.g., transcripts, proteins, intra- and extra-cellular metabolites) such that virocells are completely different from uninfected sister cells [17,18,19,20,21,28]. Thus, decades of diverse field and laboratory studies have emphasized that understanding marine viruses is critical to building predictive ecosystem, ocean, and climate models.

Despite the importance of phages and the rapidly accruing metagenomic survey-based phage genomic catalogs [29], experimentally tractable laboratory-ready phage–host model systems remain relatively few. This is particularly so for ssDNA phages, which are especially under-represented relative to dsDNA phages [30,31], and yet are found in every habitat [32], including in marine ecosystems, where they are now known to be ubiquitous and can be locally abundant [31,32,33,34,35]. Relative to dsDNA phages, ssDNA phages can differ greatly in genomic properties, encoded proteins, host recognition and attachment, host dependency during the infection cycle, replication, and lysis [32,36,37]. While mechanistically the best known ssDNA phages are arguably from the *Microviridae* family, given that phiX174 was sequenced nearly a half-century ago [38] and has been studied ever since, the diversity of ssDNA phages is greater than previously thought, and ssDNA virus taxonomy continues to expand [32,39,40]. Therefore, it is clear that additional representatives are needed in culture to better understand the mechanisms of infection of ssDNA phages, some of the invaluable players among the vast prokaryotic biosphere [41,42,43].

Here, we focus on the ecologically relevant marine *Cellulophaga baltica* host, a member of the *Flavobacteriia* class (*Bacteroidetes* phylum) that is among the most dominant organisms in marine waters [44,45] and an important contributor to carbon cycling due to degrading complex carbohydrates [45,46,47]. We leverage a collection of previously isolated and sequenced phage isolates [48,49,50,51,52] to study an ssDNA phage (phi18:4) and a dsDNA phage (phi18:1) infecting the same host (*C. baltica* strain #18). While phage genomes (through whole-genome sequencing), morphologies (through electron microscopy), and structural proteomes had been characterized [50], neither phage had been further investigated genomically or physiologically. This work adds to the collection of experimentally characterized, environmental phage–host systems, including augmenting knowledge of under-represented aquatic ssDNA phages [49], and contrasts ssDNA vs. dsDNA phage infections on the same host.

## 2. Methods

### 2.1. Accessions, Genome Reannotations, and Comparative Genomics

The genomes of all organisms are publicly available: *Cellulophaga baltica* #18 (GCF_000468615.2), phi18:1 (KC821619), and phi18:4 (KC821628). To augment previous NCBI annotations, both phage genomes were re-annotated using dram-v (v1.4.6) [53] as well as the pharokka (v1.7.3) [54] and phold (v0.2.0, https://github.com/gbouras13/phold (accessed on 19 November 2024)) pipeline, the latter being a structure-based annotation tool. Domains were also annotated using Interproscan (v5.36-75) [55]. The annotations were then manually inspected and new functions were assigned when possible (Supplementary dataset). Comparative genomics was performed using a nucleotide-based analysis (Blastn [56]) between genomes (using whole genome, fasta files) and visualized via EasyFig [57], or using an amino-acid based analysis (using GenBank files) via Clinker [58]. To generate a phylogenetic tree of all sequenced ssDNA phages, protein sequences were used in VipTree [59], which constructs proteomic trees to visualize evolutionary relationships among viruses based on whole-proteome similarities. Finally, to evaluate the ‘neighbors’ of phi18:4, ssDNA genomes belonging to the realm *Monodnaviria*, families *Finnlakeviridae*, *Alphasatellitidae*, *Anelloviridae*, *Spiraviridae* and *Tolecusatellitidae* were downloaded from RefSeq version 228. The genera *Alphapleolipovirus*, *Betapleolipovirus* and *Gammapleolipovirus*, as well as the family *Polyomaviridae* were excluded as they contained dsDNA genomes. These 2802 genomes were analyzed with vConTACT3 (https://bitbucket.org/MAVERICLab/vcontact3 (accessed on 12 February 2025)) using default parameters. Family-level placement of genomes were concordant to the ViPTree analysis, as well as for genus-level for *Flavobacterium* phage FLiP, and *Cellulophaga* phage phi12a:1, phi12:2, and Omtje1.

### 2.2. Bacterial and Phage Culture Conditions

*Cellulophaga baltica* strain #18 (Cba18) was previously isolated from the Baltic Sea [48] and grown and maintained as previously mentioned [18,48,51,52]. Briefly, cells were grown in 10 mL marine Luria–Bertani (MLB) broth (15 g L^−1^ Sigma sea salts, 0.5 g L^−1^ yeast extract, 0.5 g L^−1^ peptone, 0.5 g L^−1^ casamino acids, 3 mL glycerol) in a 125 mL Erlenmeyer flask at room temperature (RT) and without shaking. Phage lysates were produced on this host using the standard plaque assay technique [60]. Briefly, per plate, phages were diluted to achieve approximately 95% lysis in Marine Sodium Magnesium (MSM) buffer (13.4 g L^−1^ NaCl, 6.16 g L^−1^ MgSO_4_ · 7H_2_O, 3.02 g L^−1^ Tris base, pH adjust to 7–8); plated on Zobell plates (15 g L^−1^ Sigma sea salts, 1 g L^−1^ yeast extract, 5 g L^−1^ peptone, 14 g L^−1^ Difco agar); topped with 300 µL host diluted in 3.5 mL Top MSM (MSM buffer recipe with 6 g L^−1^ low melting point agarose) which was melted and cooled to 37 °C. Plates were incubated at RT for ~18–24 h. Phage lysates were collected by adding 5 mL MSM buffer per plate, shaken gently (~70 rpm) for 30 min to 3 h, 0.22 µm filtered to remove bacteria, and stored at 4 °C.

### 2.3. Adsorption Assays and One-Step Growth Curves

Adsorption assays and one-step growth curves were performed with biological triplicates. Single colonies of Cba18 were grown overnight, diluted 100-fold and grown to mid-exponential phase (>1 × 10^8^ cell mL^−1^). Cells were then diluted to 1 × 10^8^ cell mL^−1^ and independently infected with phi18:1 or phi18:4. For adsorption assays, the multiplicity of infection (MOI) was 0.3 and 0.03, respectively, to reduce instances of multiple adsorptions. Free phage (0.2 µm filtered to remove bacteria) samples were taken regularly over a 30 min period. One-step growth curves were performed similarly, except that the MOI was 0.1 and phages were allowed to adsorb for 10 min before diluting the samples 1000-fold in MLB, after which total and free phages were sampled as previously described [51] to determine latent period and burst size for each phage. Infections were determined by counting plaques after 24 and 48 h of incubation on plates (see the standard plaque assay method in the section above), and to get from plaque counts to pfu/ml, the following formula was used: PFU mL^−1^ = N × 1/DF × 1/V, where “N” is the number of plaques, “DF” is the dilution factor, and “V” is the volume of phage dilution plated. The latent period was determined as the last time point in which total and free phage abundance did not increase significantly from the previous time, as determined by a two-tailed *t*-test (*p* < 0.05), as previously performed [51,52,61]. The burst size (i.e., phages produced per infected cell) was calculated by subtracting the free phage values after the burst (i.e., point after which there was no significant increase in phage abundance) from those before the burst (averaged values from all the time points within the latent period) and then dividing that number by the number of phages that were able to infect (the difference between the total and free fraction of phages during the latent period), as previously performed [51,52,61]. Data are found in the Supplementary Dataset.

### 2.4. Chloroform Assay

To determine whether phi18:4 contains an internal lipid membrane, it was treated with 50% *v*/*v* chloroform to assess reduction in infectivity, as follows. The phage was diluted to 2 × 10^8^ PFU mL^−1^ in 0.5 mL in 6 separate tubes. Chloroform (0.5 mL) was added to 3 tubes, with the remaining 3 tubes containing no chloroform to serve as controls. Tubes were incubated at 4 °C with agitation the entire time and sampled at 5 min and 1 h by centrifuging for 2 min at 5000× *g* to separate the phases. The upper, aqueous phase was obtained and phage titer determined by plaque assay.

## 3. Results and Discussion

**Figure 1:**
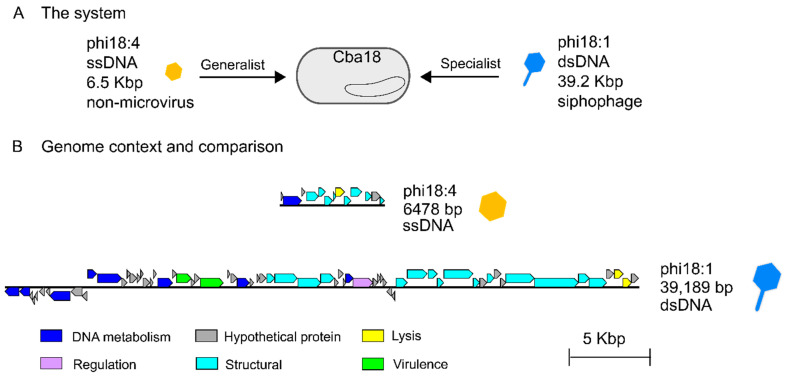
**The phages from this study and their genomes.** (**A**) Two flavophages independently infecting the same *Cellulophaga baltica* host strain #18 (Cba18), ssDNA phage phi18:4 (which is microvirus-like in morphology but not in taxonomy) and dsDNA siphophage phi18:1. (**B**) Genomic context of each phage and Blastn comparison showing lack of similarity and synteny between the genomes; generated with EasyFig [57].

Two previously isolated and sequenced phages from unrelated genera [50] were evaluated for their genomes and infection properties on the same marine *Cellulophaga baltica* host strain. Specifically, these were host *Cellulophaga baltica* strain #18 (Cba18 herein; the host used for isolating both phages [48]) and phages phi18:4 and phi18:1 that represent, respectively, a non-tailed single-stranded DNA phage with a 6.5 kbp genome and a 32 nm capsid [49] and a tailed double-stranded DNA siphophage with a 39.2 kbp genome and a ~50 nm capsid [50] (Figure 1A, Table 1). Phage phi18:4 is known to be a generalist that can infect 14 of the 21 *C. baltica* strains available [52], whereas phi18:1 is a specialist, infecting only two strains out of the 19 tested, including Cba18 with high efficiency, and another strain, Cba17, with 6 orders of magnitude lower efficiency [52]. Phages phi18:4 and phi18:1 belong to different genera [50] and share no similarities at the nucleotide or amino acid levels (Figure 1B, Table S1).

Here we studied the genomic and infection properties of phi18:4 and phi18:1 on Cba18 to establish baseline understanding of ssDNA versus dsDNA phage infections on the same host.

### 3.1. Properties of ssDNA Phage phi18:4

Due to the relative dearth of ssDNA phage genomes in the literature as compared to dsDNA phages [30,31], we first evaluated the genome of ssDNA phage phi18:4 to place it into currently understood genomic and taxonomic context. When phi18:4 was first characterized via whole-genome sequencing and electron microscopy over a decade ago, it was classified as microvirus-like [50]. More recently, however, with the discovery of additional aquatic *Bacteroidetes* ssDNA phages, phi18:4 has been proposed to belong to a new family, candidatus *Obscuriviridae* [62,63]. We confirmed that phi18:4 is indeed not a microvirus via a protein-based clustering of all sequenced ssDNA phages (Figure S1) and a homology-based comparison between phi18:4 and *Microviridae* representative phiX174 (Table S1).

**Figure 2:**
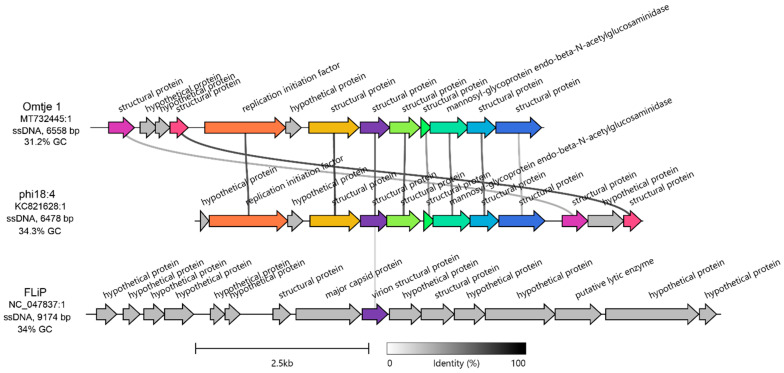
**Genomic context of phi18:4 and comparison against other ssDNA phages.** Amino acid-based gene cluster alignment of phi18:4 and Omtje (candidatus *Obscuriviridae* family) and their closest relative, FLiP (*Finnlakeviridae* family). The vertical represents amino acid percent identity, and the colors represent gene clusters. Gray genes have no similarity to other genes in the alignment. Figure generated with Clinker [58]. For nucleotide-based comparison against the rest of the ssDNA *Cellulophaga* phage public sequences in the same candidatus *Obscuriviridae* family (phi12:2, phi12a:1, and phi48:1), see Supplementary Figure S2. The genome of Omtje1 was obtained from [62] and is published under accession MT732445.1 and was rearranged to start at the 1000 bp position relative to the published sequence for this figure. The genome of FLiP was obtained from [39] and is published under accession NC_047837.

The candidatus *Obscuriviridae* family contains 4 sequenced *C. baltica* ssDNA phages (phi18:4, phi12:2, phi12a:1, and phi48:1 [50,52]) as well as Omtje (6.5 Kbp) (Figure S1), a recently discovered ssDNA phage that infects the same *Cellulophaga* genus (*Cellulophaga* sp. HaHaR_3_176 [62]). Phage phi18:4 shares > 92% identity across 100% of its genome with the other *C. baltica* ssDNA phages (which were previously identified as belonging to the same genus [50]), and 71.9% identity across 83% of its genome with Omtje (Table S2, Figure S2), including all phi18:4’s proteins with known functions (10/13; Figure 2). We then compared phi18:4 and Omtje to their closest known relative, ssDNA phage FLiP (9.2 Kbp) [62]. FLiP infects *Flavobacterium*, is the sole member of the *Finnlakeviridae* family (Figure S1), and contains a lipid membrane that is selectively obtained from its host [39,40]. A comparison of the phages in these two families, *Obscuriviridae* and *Finnlakeviridae*, revealed that phi18:4 and Omtje share one structural protein with FLiP (Figure 2). All these mentioned ssDNA phages have no other “neighbors” according to a vContact3 and a VipTree [59] analyses (Figure S1).

Next, we examined the 13 genes encoded in phi18:4’s genome–all of which are oriented in the same direction–to assess their functions (Figure 2, Figure S2). The genome is mostly composed of structural proteins (8/13) with no further annotation (even after more recent annotation efforts; see methods), except for the previously identified major capsid protein gp08 [50].

As phages with small genomes are known to rely heavily on host functions during the infection cycle [32], the remaining genes with known functions are likely host modulation proteins. Specifically, these included two proteins embedded within the structural module involved in DNA replication (replication initiation factor gp02) and in cell lysis (mannosyl-glycoprotein endo-beta-N-acetylglucosaminidase gp08), respectively (Figure 2, Figure S2). Both are present in all ssDNA phages of the same *Cellulophaga* genus [50,62], which suggests their importance for phage–host interaction biology in *Cellulophaga*. More specifically, starting with the former, the replication initiation factor is also found across other ssDNA phages, including other *Flavobacterium* ssDNA phages [64], and in plasmids [65]. It is involved in jump-starting DNA replication through the “rolling circle” replication mode by binding to the origin of replication and recruiting the host replication machinery [66,67]. As for the latter, mannosyl-glycoprotein endo-beta-N-acetylglucosaminidase belongs to the glucosaminidase protein superfamily along with homologous flagellar protein J, which hydrolyzes peptidoglycan [68,69]. This protein has also been detected in dsDNA phage genomes [70], suggesting it is not a unique feature of ssDNA phages. Since mass spectrometry-based proteomic measurements of purified viral particles suggested that gp08 is contained inside the phi18:4 virion [50], it may aid not only in cell lysis, but also in injecting DNA into the cell through host peptidoglycan degradation, similar to dsDNA phage PM2 which also contains an internal membrane [71].

Finally, because several aquatic ssDNA phages have now been found to contain lipid membranes in their virions [39,64], we hypothesized that phi18:4 contains an internal lipid membrane. To test this hypothesis, we treated phi18:4 with chloroform, which reduces infectivity of lipid-containing phages [72]. This experiment revealed that after a 5 min incubation, viral particles were >100-fold less infective, and after 1 h incubation there were no detectably infective particles remaining (Table S2). These data suggest that phi18:4 has an internal lipid membrane and, based on knowledge from other lipid-containing phages, this internal lipid would likely play critical roles at multiple steps of the infection cycle, from viral genome ejection to assembly and lysis [72,73].

### 3.2. Properties of dsDNA Phage phi18:1

**Figure 3:**
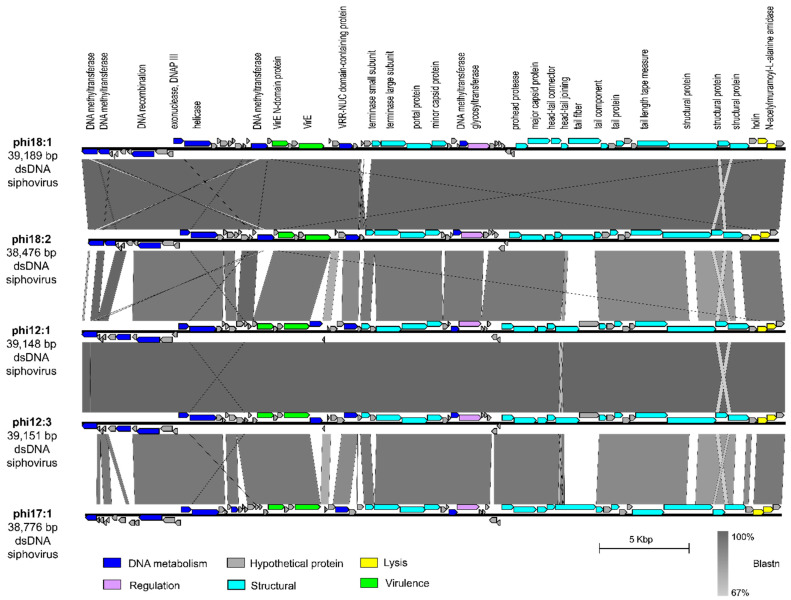
**Comparison of dsDNA phage phi18:1 with the other phages in the same genus.** Genes are color-coded by the functional category. Annotations are added for every protein with a known function. Synteny is shown through Blastn. Figure generated with EasyFig [57]. All phages infect *Cellulophaga baltica*. Genomes were obtained from a prior study [50] and can be found under accessions NC_021790.1 (phi18:1), KC821627 (phi18:2), NC_021791 (phi12:1), KC821615 (phi12:3), and NC_021795 (phi17:1).

We next evaluated phage phi18:1 because it also infects Cba18 but is contrastingly different to phi18:4 in genome architecture and host range. The dsDNA genome of phi18:1 encodes 67 proteins broadly organized into a DNA metabolism module, a structural module, and a lysis module (Figure 1B and Figure 3). This phage does not contain an RNA polymerase (RNAP) or translation-related genes (including tRNAs), as myriad other dsDNA phages do [74,75,76]; therefore, it must rely on the host’s transcription and translation machinery. However, it does contain several genes involved in DNA replication, including one involved in DNA recombination (gp08), a helicase (gp12), and the exonuclease subunit of DNA polymerase III (gp11). This last one is involved in proofreading DNA replication in *E. coli*, which enables tight control of DNA polymerization and fidelity [77].

Additionally, phi18:1 also encodes *four* DNA methyltransferases (MTases); three within the DNA replication module and one within the structural module (Figure 3). These genes are best known for their roles in methylating phage DNA to protect against host restriction enzymes [78]. The presence of multiple MTases has been associated with phages with broad host ranges to defend against restriction enzymes from different hosts [78]. Alternatively, another explanation for encoding multiple MTases that are dispersed throughout the genome is that these genes are involved in other functions during the infection cycle beyond DNA protection, including DNA replication, transcriptional regulation, or DNA packaging [78]. As phi18:1 has a narrow host range, we posit that phi18:1’s MTases are involved in DNA protection as well as additional regulatory roles during the infection cycle.

The phi18:1 genome contained three additional genes not involved in DNA metabolism, structure, or lysis: two virulence-related genes (*virE*; gp23 and gp26) at the end of the DNA metabolism module, and a glycosyltransferase (gp41) within the structural module (Figure 3). Regarding the former, the presence of *virE* genes among DNA metabolism genes is common in *Staphylococcus aureus* virulent and temperate phages where there is high recombination between phages (virulent or temperate) and host [79,80]. More broadly, virulence factors and toxins are commonly carried by temperate phages across environments, including aquatic, even if the host is not a pathogen, and contribute to host pathogenicity and the evolution of diseases [79,81,82,83]. While surveying the genomes of other *C. baltica* phages [50] we found 1–2 copies of these virulence genes in phages of the same or different genus as phi18:1 (Figures S3 and S4; Table S3), therefore suggesting high recombination between *C. baltica* and phages.

Finally, glycosyltransferase gene gp41 was also found in all phages of the same genus as phi18:1 (Figure 3), but in none of the other sequenced *C. baltica* phages [50] (Table S3). Glycosyltransferases are members of the Carbohydrate-Active EnZymes (CAZy) that transfer sugar moieties to a variety of substrates, including DNA, proteins, and lipids [84]. They have been found in *Shigella* and *E. coli*-infecting phages, where they modify bacterial lipopolysaccharides (LPS) [85,86] or phage genomes [87,88] for protection against restriction endonucleases, respectively. Presence in specialist phages like phi18:1 suggests this gene could either aid in LPS modification to provide superinfection exclusion in Cba18—which is a strategy commonly observed for prophages [89], provide another layer of phage DNA protection against host restriction systems as in the T-even *E. coli* phages [87,88], or have regulatory roles by altering other biomolecules like proteins or lipids [90].

### 3.3. Infection Properties of the ssDNA and the dsDNA Phage on the Same Strain

**Figure 4:**
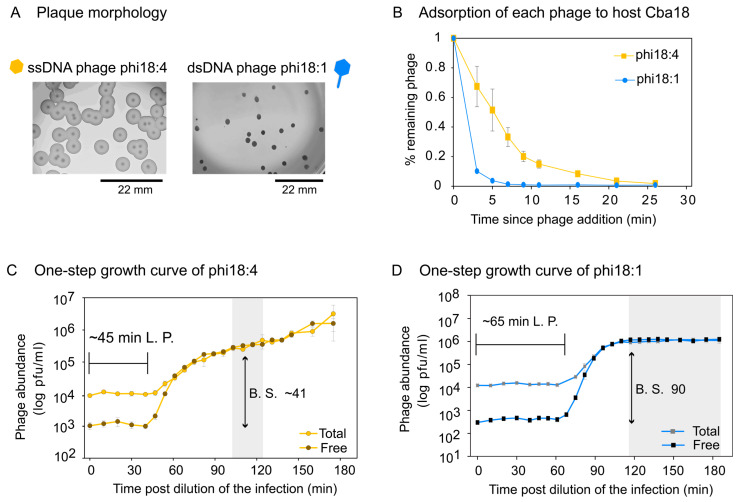
**Phage infection properties on *Cellulophaga baltica* #18 (Cba18).** (**A**) Plaque assays showing plaque morphology of each phage on Cba18 with a scale bar. Phi18:4 has a clear center with a turbid, temperate phage-like surrounding, while phi18:1 has a smaller, clear plaque with no turbidity. None of the phages have identifiable lysogeny genes. (**B**) Adsorption dynamics of phi18:4 and phi18:1 from independent infections on Cba18 and a multiplicity of infection (MOI) < 0.4. (**C**) One-step growth curve of phi18:4 on Cba18. (**D**) One-step growth curve of phi18:1 on Cba18. For both C and D, MOI is 0.1, total and free phage concentrations are plotted (average and standard error from three biological replicates), and calculated latent period (L.P.) and burst size (B.S.) estimates are indicated. Shaded in gray is the estimated end of the first burst. Data can be found in the Supplementary Dataset.

We next evaluated the independent infections of ssDNA phage phi18:4 and dsDNA phage phi18:1 on Cba18. Plaque assays revealed different plaque morphologies whereby phi18:4 has a big plaque with a clear center and a turbid surrounding, and phi18:1 has a smaller, clear plaque with no turbidity (Figure 4A).

Adsorption assays for phi18:4 and phi18:1 on Cba18 revealed that 85% and 96% of originally added phi18:4 and phi18:1 phages had, respectively, adsorbed to Cba18 by 11 min and 5 min (Figure 4B). Comparing against a phage from a different genus that is known to infect this same host *inefficiently* (generalist dsDNA podophage phi38:1), for which only 68% of added phages have adsorbed by 30 min [51], phages phi18:4 and phi18:1 adsorb efficiently to Cba18.

One-step growth curves for phi18:4 and phi18:1 on Cba18 revealed that phi18:4 displayed a latent period of ~45 min and a burst size of ~41 (Figure 4C), whereas phi18:1 displayed a latent period of ~65 min and a burst size of ~90 (Figure 4D). For ssDNA phages, lysis time (i.e., latent period) is thought to depend solely on the growth rate of the host because of lacking the same lysis machinery found in dsDNA phages used to time cell burst (i.e., membrane holin and peptidoglycan hydrolase; [32]). Additionally, theory predicts that high host densities would select for shorter latent periods [91,92,93,94], and that there is a trade-off between latent period and burst size [95,96,97]. Thus, different host growth conditions could presumably change the outcome of these infections.

We then compared these infections to the only other phage–host interactions that have been published in *C. baltica* under similar growth conditions [51,52,61]. Even with a small sample size of 5 additional flavophages studied in Cba18, this comparison revealed that infection dynamics on this host is highly variable depending on the phage, with latent periods and burst sizes ranging from 45 to 300 and 1 to 90, respectively (averages of 138.3 min ±105 and 32.1 ± 31.4 for latent period and burst size, respectively) (Table S4). Among them, phi18:4 displayed the shortest latent period (Figure S5A) and phi18:1 the largest burst size (Figure S5B) Further, comparing again against inefficiently infecting dsDNA podophage phi38:1, whose latent period is ~11 h via plaque assays or 5 h via phageFISH [61], the one-step growth curves supported the adsorption data in that, relative to phi38:1, phi18:4 and phi18:1 infect Cba18 efficiently.

Together, these data promote knowledge on marine phage ecology by describing the genomic properties and infection characteristics of a dsDNA and an ssDNA phage on the same host. In general, marine phages are known to alter bacterial composition and evolution through infection, as well as nutrient cycling through the shunt (biomass is redirected away from grazers and towards bacteria) and shuttle (virus-infected cells induce aggregates that promote biomass sinking) hypotheses [22,98,99]. This in turn affects the fate of carbon, with viruses now recognized to be better predictors of carbon export to the deep oceans than microbes [24]. Additionally, marine phages contain a large repertoire of auxiliary metabolic genes [100] that metabolically reprogram their hosts during infection and, through transforming cells into new entities (called virocells [101]), alter the transcript, protein, and metabolite pool of the marine ecosystem [19,21,102], which in turn have impacts on cross-kingdom interactions [20,23]. However, as this knowledge is largely based on dsDNA and not on ssDNA phages, or on one phage type infecting one host at a time, future work can focus on examining the impacts of ssDNA versus dsDNA virocells to better understand the contribution of these different phages and their virocells to marine ecology.

## 4. Conclusions

Mechanistic knowledge of ssDNA phages has arguably derived from two families [37], *Microviridae* (with phiX174 as its representative) and *Inoviridae* (including the filamentous Ff phages) [32]. Exploration of the virosphere in the past few years has revealed that the diversity of ssDNA phages is greater than previously thought [39,62]. However, dsDNA phages are still the easiest to isolate and therefore represent the majority of phage–host model systems available [32]. Yet, as ssDNA and dsDNA phages differ greatly, understanding mechanisms of phage infection and leveraging this knowledge for biotechnological applications—including, but not limited to, phage therapy [37,103]—requires increasing the diversity of known environmental phage–host interactions, such as contrasting ssDNA with dsDNA phages on the same host, given that hosts are not necessarily infected by just one phage type in nature. Here, we leveraged previously sequenced and morphologically characterized phages from ssDNA and dsDNA genomes and studied their infections on the same environmental *Bacteroidetes* host (marine *Cellulophaga baltica*). We reveal that despite the very different genomic architectures and host-ranges, both phages infect in a similar efficient manner. Given this and the fact that smaller phages (i.e., here, phi18:4) heavily rely on host machinery due to their limited coding capacity [32], mechanistic characterization of the different strategies adopted by each of these phages to infect the same host will be an interesting future endeavor. Finally, with increasing knowledge of the variety of phage infection lifestyles for dsDNA phages—from lytic to lysogenic to cryptic [104]—and their biological importance in nature [105], it will be equally important to explore the infection continuum for the world of ssDNA phages.

## Figures and Tables

**Table 1:** The phages used in this study, their genomic characteristics, and infection details on host Cba18.

Phage	Family	Genus	Genome	Morphology	Genome Size (kb)	ORFs	%GC	Host Range	% Adsorbed Phages	Latent Period	Burst Size	Infection Efficiency
phi18:4	Obscuriviridae ^1^	Cebaduodecimvirus	ssDNA	Microvirus-like	6.5	13	34.3	Broad ^2^	80% (by 10 min)	~45 min	~41	Efficient
phi18:1	Unclassified	Helsingorvirus	dsDNA	siphovirus	39.2	65	36.5	Narrow ^2^	99% (by 10 min)	~65 min	~90	Efficient

^1^ Proposed by [62] and under consideration by the ICTV. ^2^ Generalist phages have broad host ranges and specialist phages have narrow host ranges when considering different strains of *C. baltica*, as described in [52].

## Data Availability

Data are available at the supplementary dataset and Supplementary Materials.

## Supplementary Materials

The following supporting information can be downloaded at: https://www.mdpi.com/article/10.3390/v17030365/s1, Figure S1: Placement of phi18:4 in the context of all sequenced ssDNA viruses. (A) VipTree-generated dendrogram of ssDNA viruses present in RefSeq as of January 2025, and Omtje1, showing the clustering of phi18:4 into a new family with Omtje. VipTree generates a "proteomic tree" of viral genomes based on genome-wide similarity computed by tBLASTx [59]. Similar to previously presented data [62]. (B) Cytoscape network of nodes (viruses) and their shared genes (edges) between ph18:4 and related viruses sharing clusters as defined by vConTACT3. Edge thickness indicates the number of shared genes (also labeled), with physical distance between genomes indicated by their weight (distance – 1). For visual clarity, Omtje (distance: 0.93) was moved closer to the rest of the cluster (average distance: 0.11). (C) vConTACT3 protein cluster (PC) profile of protein clusters shared at 30% minimum clustering identity between phi18: 4 related sequences present in RefSeq as of January 2025 as well as Omtje. Figure S2: Genomic similarity between candidatus *Obscuriviridae* ssDNA flavophages. Blastn comparison between the *Cellulophaga* phages previously sequenced [50,62]. Omtje1 was chosen among the Omtje representatives since they are all highly similar [62]. Figure generated with EasyFig [57]. Phage genomes can be found under accessions MT732445.1 (Omtje 1), KC821628 (phi18:4), KC821631 (phi48:1), NC_021805 (phi12a:1), and NC_021797 (phi12:2). Blastn results are also in Table S1. Figure S3: Comparison between *Cellulophaga baltica* siphophage phi18:1 and five myophages from the *CbaSMlikevirus* genus. All phages infect *C. baltica*. Phage phi18:1 (NC_021790.1) with narrow host range, which was previously proposed to belong to the *Cba181likevirus* genus [50] and is currently classified as belonging to the *Helsingorvirus* genus by ICTV, is compared via tBlastx against dsDNA phages with broad host ranges phiSM (KC821616), phi3:1 (KC821630), phi3ST:2 (KC821610), phi38:2 (KC821629), and phi47:1 (KC821634) previously discovered and designated to the *CbaSMlikevirus* genus [50]. Current ICTV taxonomy for those five phages does not include a genus designation. Colors for phi18:1 are the same as in Figure 1 and Figure 3, and for the other phages genes are in orange except the virulence factor which is highlighted in green. Figure created via EasyFig [57]. Figure S4: Comparison between *Cellulophaga baltica* siphophages phi18:1 and the three in the *Cbastvirus* genus. All phages infect *C. baltica*. Phage phi18:1 (NC_021790.1) has a narrow host range, was previously proposed to belong to the *Cba181likevirus* genus [50], and is currently classified as belonging to the *Helsingorvirus* genus by ICTV. It is compared via tBlastx against dsDNA phages with broad host ranges phi13:1 (KC821625), phiST (NC_020842), and phi19:2 (KC821621) previously discovered and designated to the *Cba131likevirus* genus [50]. Current ICTV taxonomy for those three phages assigns them to the *Cbastvirus* genus. Colors for phi18:1 are the same as in Figure 1 and Figure 3, and for the other phages genes are in orange except the virulence factor which is highlighted in green. Figure created via EasyFig [57]. Figure S5: Published phage infection dynamics on *Cellulophaga baltica* host strain #18 (Cba18). (A) Latent period and (B) burst size of all published phage—host interactions on Cba18 all under similar laboratory growth conditions. Number of biological replicates used for each are in parenthesis. Data and phages obtained from [51,52,61]. Superscripts refer to ^1^ [52], ^2^ [51], and ^3^ [61]. Table S1: Genomic comparison between phages. Omtje 1 was chosen as representative of all isolated, Omtje-like ssDNA flavophages published [62]. Table S2: Incubation of phi18:4 with (+) and without (-) chloroform (50% v/v) for 5 min and 1 h. Infectivity is represented by plaque counts (plus/minus standard deviation) using Cba18 as host for biological duplicates, for different dilution factors. The “-“ represents no information (i.e., that sample was not plated in that dilution factor). Table S3: Select phage genes and their presence in the published *Cellulophaga baltica* phages. Genomic information for the phages was obtained from the original publication [50]. Table S4: The published phage infections on *Cellulophaga baltica* host strain #18.
