# Supplementary material for: Infection and Genomic Properties of Single- and Double-Stranded DNA Cellulophaga Phages
Source: Viruses. 2025 Mar 3;17(3):365. doi: 10.3390/v17030365 (PMC11946311; doi:10.3390/v17030365)
Supplement: Supplementary file 1 [file viruses-17-00365-s001.zip › viruses-3507787-Supplementary Materials-to XML.pdf]

Supplementary Materials

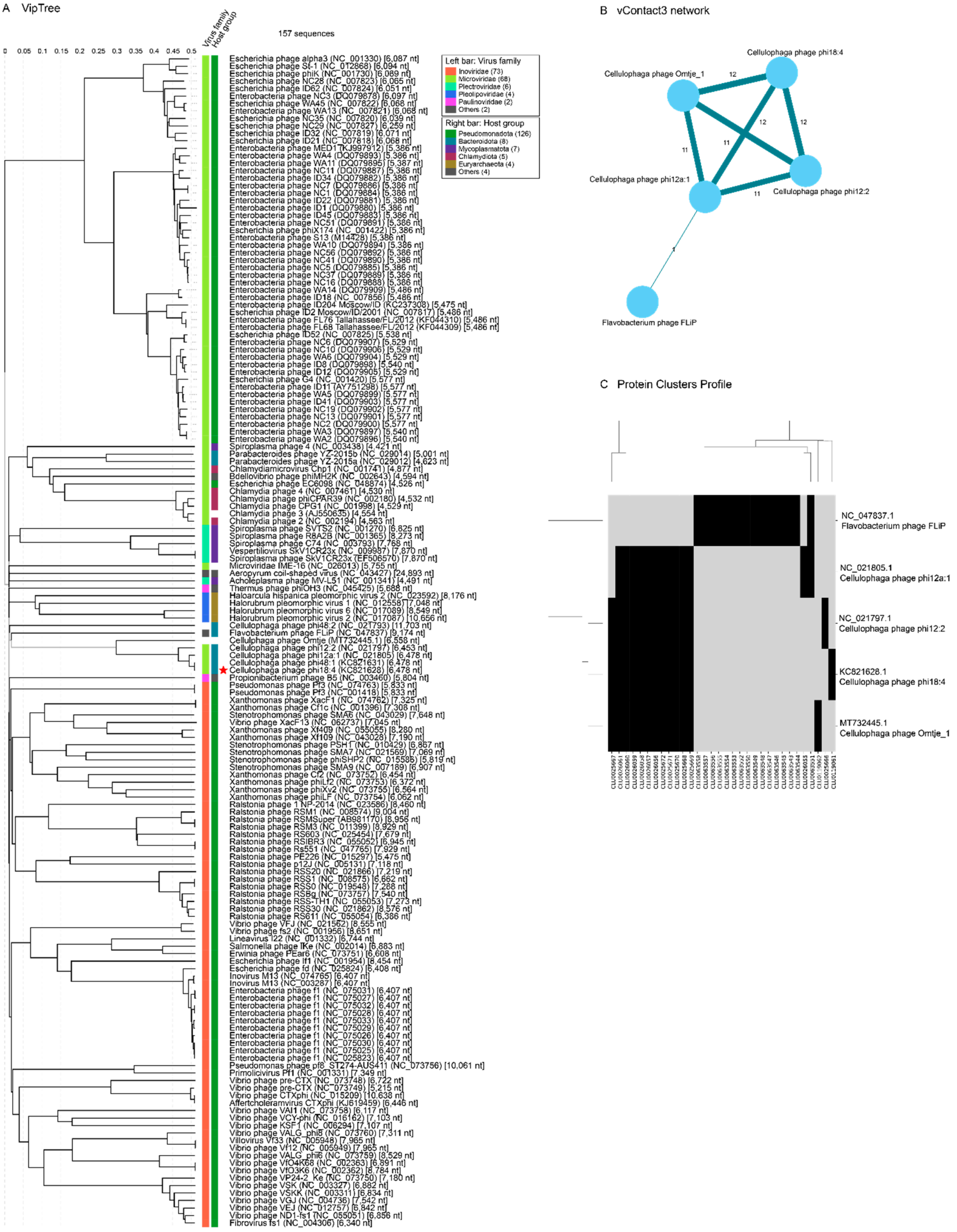

Figure S1: Placement of phi18:4 in the context of all sequenced ssDNA viruses. (A) VipTree-generated dendrogram of ssDNA viruses present in RefSeq as of January 2025, and Omtje1, showing the clustering of phi18:4 into a new family with Omtje. VipTree generates a "proteomic tree" of viral genomes based on genome-wide similarity computed by tBLASTx [59]. Similar to previously presented data [62]. (B) Cytoscape network of nodes (viruses) and their shared genes (edges) between ph18:4 and related viruses sharing clusters as defined by vCon-TACT3. Edge thickness indicates the number of shared genes (also labeled), with physical distance between genomes indicated by their weight (distance – 1). For visual clarity, Omtje (distance: 0.93) was moved closer to the rest of the cluster (average distance: 0.11). (C) vConTACT3 protein cluster (PC) profile of protein clusters shared at 30% minimum clustering identity between phi18: 4 related sequences present in RefSeq as of January 2025 as well as Omtje.

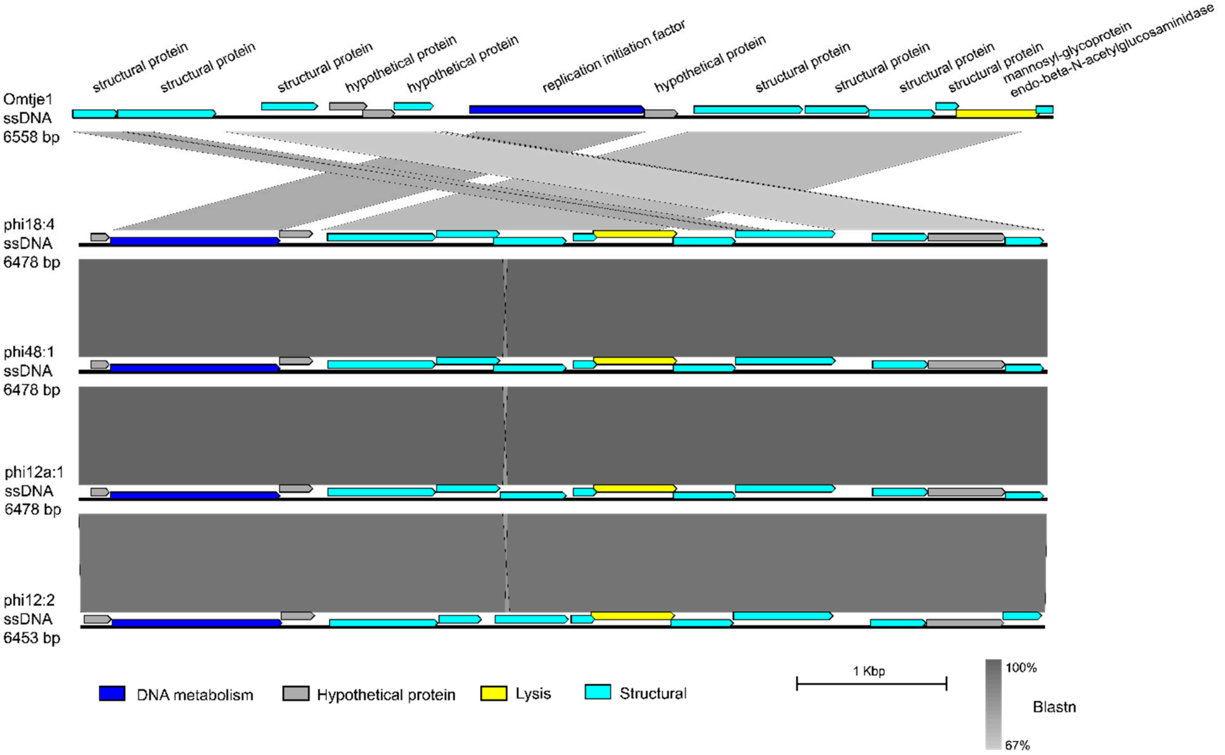

Figure S2: Genomic similarity between candidatus *Obscuriviridae* ssDNA flavophages. Blastn comparison between the Cellulophaga phages previously sequenced [50,62]. Omtje1 was chosen among the Omtje representatives since they are all highly similar [62]. Figure generated with EasyFig [57]. Phage genomes can be found under accessions MT732445.1 (Omtje 1), KC821628 (phi18:4), KC821631 (phi48:1), NC_021805 (phi12a:1), and NC_021797 (phi12:2). Blastn results are also in Table S1.

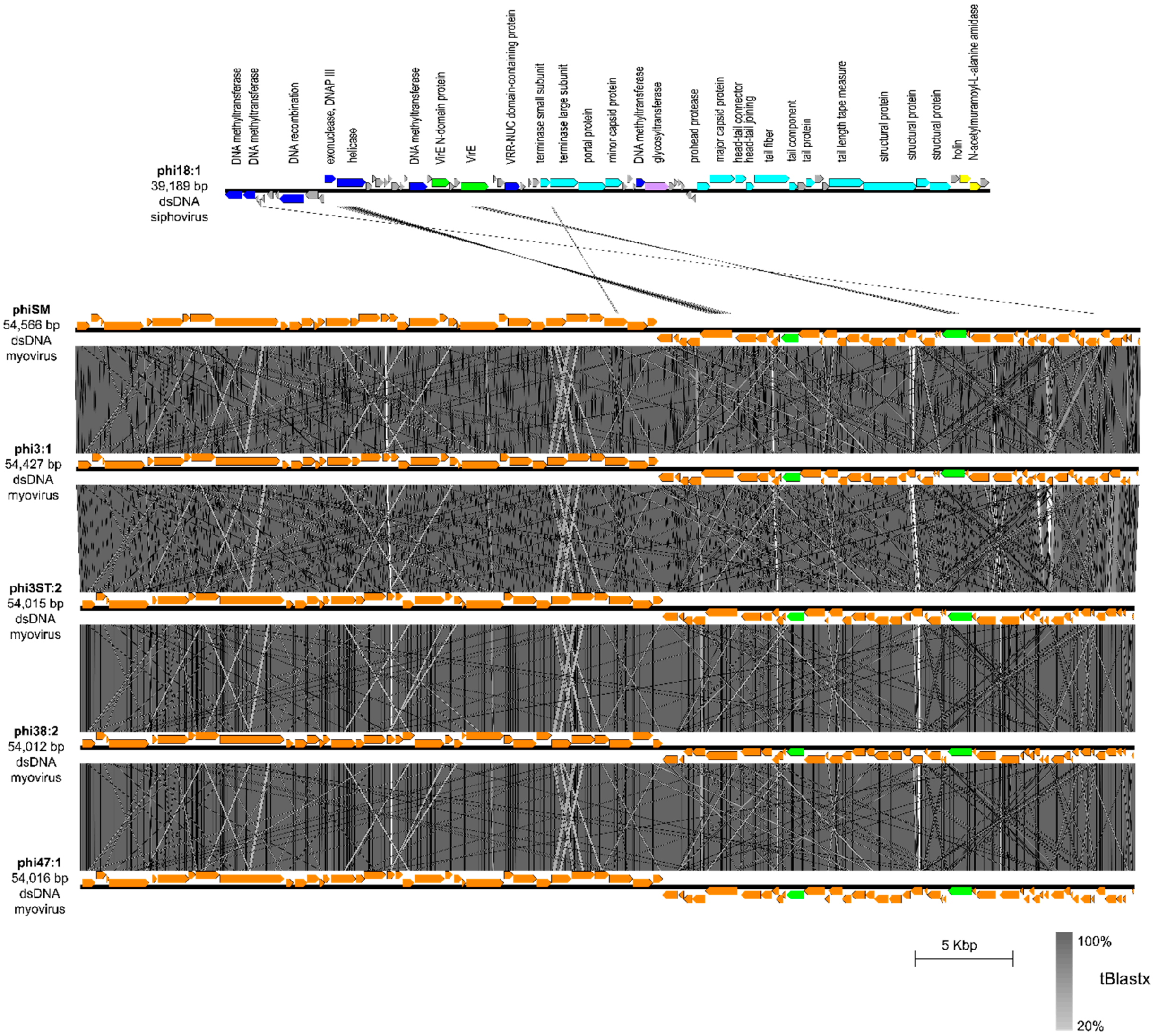

Figure S3: Comparison between *Cellulophaga baltica* siphophage phi18:1 and five myophages from the *CbaSMlikevirus* genus. All phages infect C. baltica. Phage phi18:1 (NC_021790.1) with narrow host range, which was pre-viously proposed to belong to the Cba181likevirus genus [50] and is currently classified as belonging to the Helsingorvirus genus by ICTV, is compared via tBlastx against dsDNA phages with broad host ranges phiSM (KC821616), phi3:1 (KC821630), phi3ST:2 (KC821610), phi38:2 (KC821629), and phi47:1 (KC821634) previously discovered and designated to the CbaSMlikevirus genus [50]. Cur-rent ICTV taxonomy for those five phages does not include a genus designation. Colors for phi18:1 are the same as in figures 1 and 3, and for the other phages genes are in orange except the virulence factor which is highlighted in green. Figure created via EasyFig [57].

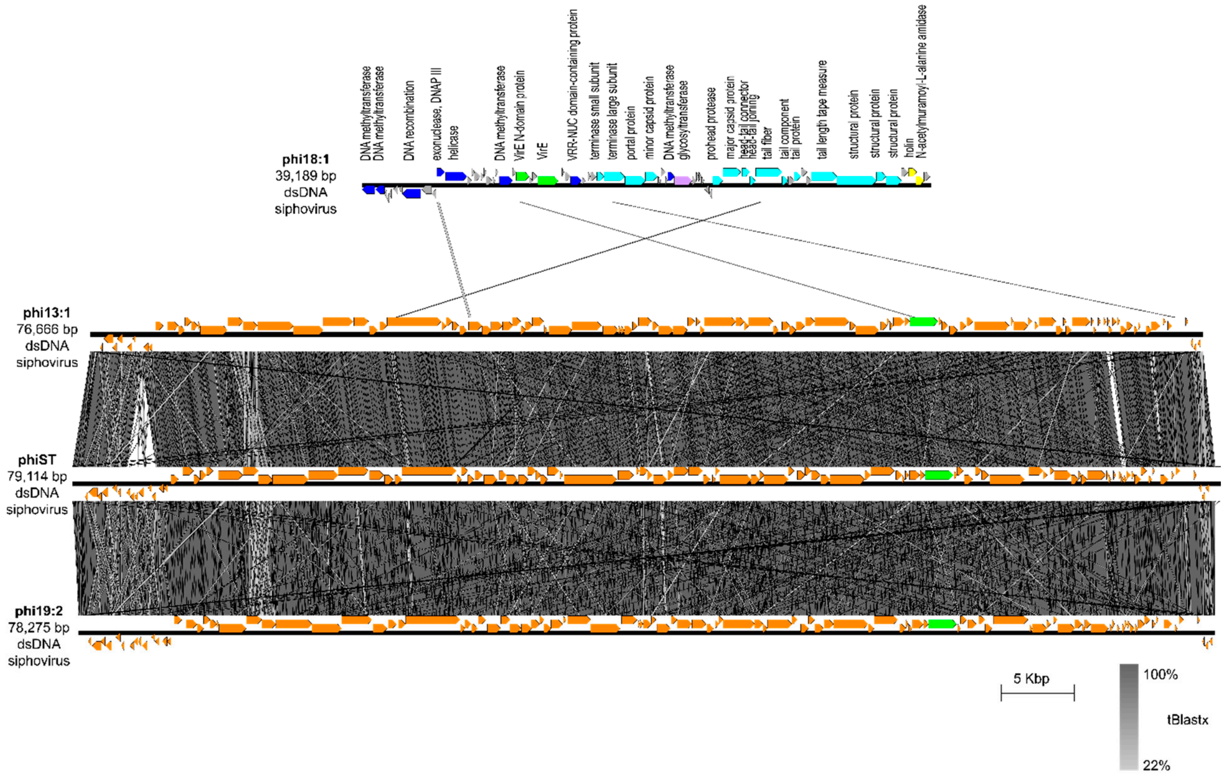

Figure S4: Comparison between *Cellulophaga baltica* siphophages phi18:1 and the three in the *Cbastvirus* genus. All phages infect C. baltica. Phage phi18:1 (NC_021790.1) has a narrow host range, was previously proposed to belong to the Cba181likevirus genus [50], and is currently classified as belonging to the Helsingorvirus genus by ICTV. It is compared via tBlastx against dsDNA phages with broad host ranges phi13:1 (KC821625), phiST (NC_020842), and phi19:2 (KC821621) previously discovered and designated to the Cba131likevirus genus [50]. Current ICTV taxonomy for those three phages as-signs them to the Cbastvirus genus. Colors for phi18:1 are the same as in figures 1 and 3, and for the other phages genes are in orange except the virulence factor which is highlighted in green. Figure created via EasyFig [57].

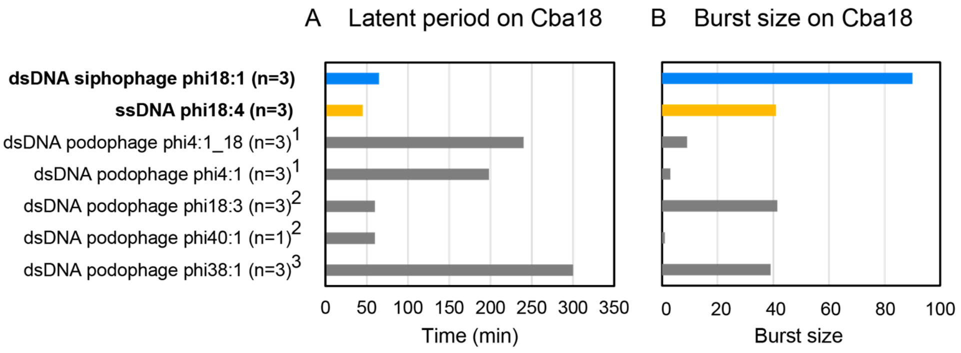

Figure S5: Published phage infection dynamics on *Cellulophaga baltica* host strain #18 (Cba18). (A) Latent period and (B) burst size of all published phage—host interactions

on Cba18 all under similar laboratory growth conditions. Number of biological replicates used for each are in paren-thesis. Data and phages obtained from [51,52,61]. Superscripts refer to [1][52], [2][51], and [3][61].

Table S1: Genomic comparison between phages. Omtje 1 was chosen as representative of all isolated, Omtje-like ssDNA flavophages published [62].

Analysis	Comparison	Max score	Total score	Query coverage	E-value	Percent identity
Blastn	phi18:4 vs phi48:1	11629	11775	100%	0	99.81%
Blastn	phi18:4 vs phi12a:1	11007	11144	100%	0	97.68%
Blastn	phi18:4 vs phi12:2	9309	9450	100%	0	91.84%
Blastn	phi18:4 vs Omtje 1	1210	2861	83%	0	71.86%
Blastn	phi18:4 vs Flip	34.6	64.8	1%	0.004	80.56%
Bastn	phi18:4 vs phiX174	NA	NA	NA	NA	NA
Blastn	phi18:4 vs phi18:1	NA	NA	NA	NA	NA
Blastn	phi18:4 vs phi18:3	NA	NA	NA	NA	NA
Blastn	phi18:1 vs phi18:3	172	521	2%	3.00E-43	73.68%
Blastn	phi18:1 vs phi18:2	41569	70546	99%	0.00E+00	100.00%
Blastn	phi18:1 vs phi12:1	7743	47431	83%	0.00E+00	94.28%
Blastn	phi18:1 vs phi12:3	7736	47441	83%	0.00E+00	94.26%
Blastn	phi18:1 vs phi17:1	12450	54172	87%	0.00E+00	96.05%

Table S2: Incubation of phi18:4 with (+) and without (-) chloroform (50% v/v) for 5 min and 1 h. Infectivity is represented by plaque counts (plus/minus standard deviation) using Cba18 as host for biological duplicates, for different dilution factors. The "-" represents no information (i.e. that sample was not plated in that dilution factor).

	5 min incubation		1 h incubation	
Dilution factor	+Chloroform	-Chloroform	+Chloroform	-Chloroform
None	-	-	0	-
1:100	-	-	0	-
1:1000	0	-	-	-
1:10000	0	-	-	-
1:100000	-	120±16	-	120.3±4
1:1000000	-	18±8	-	15±2
Loss in infectivity	At least 100-fold reduction		100% loss of infectivity	

Table S3: Select phage genes and their presence in the published *Cellulophaga baltica* phages. Genomic in-formation for the phages was obtained from the original publication [50].

DNA type	Genus	Family	Host range (Narrow	Phage	DNA methyltransf erase (MTase)	mannosyl-glycoprotein endo-beta-N-	replicat ion initiatio n factor	virulen ce	glycosyltransf erase

			: 1-3 hosts)			acetylglucosaminidase			
**dsDNA**	**Cba181like**	**sipho**	**Narrow**	**phi18:1**	4	0	0	2	1
dsDNA	Cba181like	sipho	Narrow	phi12:1	5	0	0	2	1
dsDNA	Cba181like	sipho	Narrow	phi12:3	4	0	0	2	1
dsDNA	Cba181like	sipho	Narrow	phi17:1	2	0	0	2	1
dsDNA	Cba181like	sipho	Narrow	phi18:2	4	0	0	2	1
**ssDNA**	**Cba184like**	**non-microvirus**	**Broad**	**phi18:4**	0	1	1	0	0
ssDNA	Cba184like	microviridae	Broad	phi12:2	0	1	1	0	0
ssDNA	Cba184like	microviridae	Broad	phi12a:1	0	1	1	0	0
ssDNA	Cba184like	microviridae	Broad	phi48:1	0	1	1	0	0
dsDNA	Cba401like	podo	Broad	phi38:1	0	0	0	0	0
dsDNA	Cba401like	podo	Broad	phi40:1	0	0	0	0	0
dsDNA	Cba183like	podo	Narrow	phi13:2	1	0	0	0	0
dsDNA	Cba183like	podo	Narrow	phi18:3	3	0	0	0	0
dsDNA	Cba183like	podo	Narrow (but infects 1 strain very well and 5 others less well)	phi19:3	0	0	0	0	0
dsDNA	Cba183like	podo	Narrow (but infects 1 strain well and 3 others not so well)	phi46:3	1	0	0	0	0
dsDNA	Cba142like	podo	Narrow	phi14:2	0	0	0	0	0
dsDNA	Cba41like	podo	Broad	phi4:1	3	0	0	0	0
dsDNA	Cba41like	podo	Broad	phi17:2	3	0	0	0	0
dsDNA	CbaSMlike	myo	Broad	phiSM	0	0	0	2	0
dsDNA	CbaSMlike	myo	Broad	phi3:1	0	0	0	2	0

dsDNA	CbaSMlike	myo	Broad	phi3ST:2	0	0	0	2	0
dsDNA	CbaSMlike	myo	Broad	phi38:2	0	0	0	2	0
dsDNA	CbaSMlike	myo	Broad	phi47:1	0	0	0	2	0
dsDNA	Cba391like	sipho	Broad	phi39:1	0	0	0	0	0
dsDNA	Cba461like	sipho	Broad	phi46:1	0	0	0	1	0
dsDNA	Cba101like	sipho	Narrow	phi10:1	2	0	0	0	0
dsDNA	Cba101like	sipho	Narrow	phi19:1	1	0	0	0	0
dsDNA	Cba131like	sipho	Broad	phi13:1	0	0	0	1	0
dsDNA	Cba131like	sipho	Broad	phiST	0	0	0	1	0
dsDNA	Cba131like	sipho	Broad	phi19:2	0	0	0	1	0
ssDNA	Cba482like	novel	Broad	phi48:2	0	0	0	0	0

Table S4: The published phage infections on *Cellulophaga baltica* host strain #18.

phage genome, morphology	phage--host pair	Phage genome size (Kbp)	Efficient/inefficient	Biological replicates	latent period (min)	burst size	Growth parameters	Generalist or specialist?	Reference
dsDNA, podovirus	phi38:1 on Cba18	72.5	Inefficient	3	300	39	Rich medium and room temperature	Generalist	Dang & Howard-Varona 2015
dsDNA, podovirus	phi40:1 on Cba18	72.5	?	1	60	1	Rich medium and room temperature	Generalist	Holmfeldt & Howard-Varona 2014
dsDNA, podovirus	phi18:3 on Cba18	71.4	Efficient	3	60	41.4	Rich medium and room temperature	Specialist	Holmfeldt & Howard-Varona 2014
dsDNA, podovirus	phi4:1 on Cba18	145.9	?	3	198	3	Rich medium and room temperature	Generalist	Holmfeldt 2016
dsDNA, podovirus	phi4:1_18 on Cba18	145.9	?	3	240	9	Rich medium and room temperature	Generalist	Holmfeldt 2016
ssDNA, microvirus-like	phi18:4 on Cba18	6.5	Efficient	3	45	41	Rich medium and room temperature	Generalist	This study
dsDNA, siphovirus	phi18:1 on Cba18	39.2	Efficient	3	65	90	Rich medium and room temperature	Specialist	This study
